# The Hydrolysis Rate of Paraoxonase-1 Q and R Isoenzymes: An In Silico Study Based on In Vitro Data

**DOI:** 10.3390/molecules27206780

**Published:** 2022-10-11

**Authors:** Sedat Karabulut, Basel Mansour, Gerardo M. Casanola-Martin, Bakhtiyor Rasulev, James W. Gauld

**Affiliations:** 1 Department of Chemistry and Biochemistry, University of Windsor, Windsor, ON N9B 3P4, Canada; 2 Department of Coatings and Polymeric Materials, North Dakota State University, 1735 NDSU Research Park Dr, Fargo, ND 58102, USA

**Keywords:** PON1, RPON1, isoenzymes, molecular modeling, molecular docking, molecular dynamics, QM/MM, QSAR

## Abstract

Human serum paraoxonase-1 (PON1) is an important hydrolase-type enzyme found in numerous tissues. Notably, it can exist in two isozyme-forms, Q and R, that exhibit different activities. This study presents an in silico (QSAR, Docking, MD and QM/MM) study of a set of compounds on the activity towards the PON1 isoenzymes (QPON1 and RPON1). Different rates of reaction for the Q and R isoenzymes were analyzed by modelling the effect of Q192R mutation on active sites. It was concluded that the Q192R mutation is not even close to the active site, while it is still changing the geometry of it. Using the combined genetic algorithm with multiple linear regression (GA-MLR) technique, several QSAR models were developed and relative activity rates of the isozymes of PON1 explained. From these, two QSAR models were selected, one each for the QPON1 and RPON1. Best selected models are four-variable MLR models for both Q and R isozymes with squared correlation coefficient R^2^ values of 0.87 and 0.83, respectively. In addition, the applicability domain of the models was analyzed based on the Williams plot. The results were discussed in the light of the main factors that influence the hydrolysis activity of the PON1 isozymes.

## 1. Introduction

Human serum Paraoxonase-1 (PON1) is a physiologically important Ca^2+^-dependent hydrolase enzyme. Indeed, it is widely distributed among tissues, such as liver, kidney, intestine and serum, and can hydrolyze a variety of substrates [1,2]. PON1 is biosynthesized mainly in the liver and secreted into the blood where it associates predominantly with high-density lipoprotein (HDL) [3,4,5,6], due to a hydrophobic signal sequence in its N-terminal region [7]. PON1 isoforms are known to have a broad range of roles including being involved in mediating against cardiovascular diseases, drug metabolism and bacterial infections [8,9]. These roles reflect the fact that it can hydrolyze a wide variety of lactones, thiolactones, aryl esters, cyclic carbonates, organophosphorus compounds and estrogen esters [10,11,12]. For instance, one group of potentially important lactone substrates is the statins, which inhibit cellular cholesterol synthesis and are widely used to reduce LDL-cholesterol to prevent cardiovascular diseases [2,7]. Meanwhile, PON1 can also degrade several therapeutic drugs, organophosphorus compound pesticides, as well as nerve gases such as Sarin and Soman [13,14]. Indeed, PON1 was first described in the 1940s when Mazur observed that mammalian tissues exhibited enzymatic activity capable of hydrolyzing organophosphate pesticides [15]. The enzymes were further classified by Norman Aldridge as “A”-esterase if they were capable of hydrolyzing organophosphorus compounds, as opposed to “B”-esterase which are inhibited by organophosphorus compounds [16]. Ultimately, the common use of the organophosphorus compound derivative paraoxon as a substrate for the enzyme led to the universal adoption of the name Paraoxonase [13].

**Figure 1:**
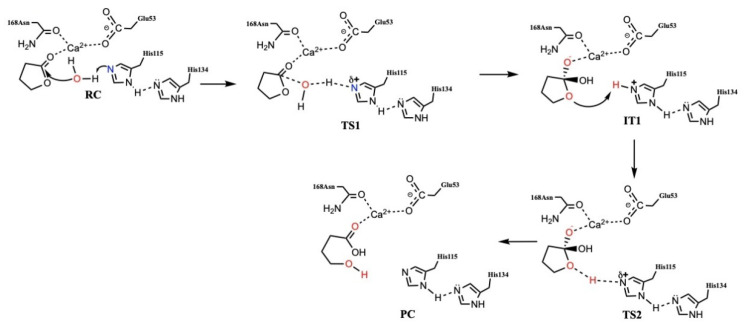
One of the proposed [18,19] catalytic mechanisms of PON1 (RC = Reactant complex; TS = Transition State; IT = Intermediate; and PC = Product Complex).

Based on structures obtained as part of X-ray crystallographic studies on PON1, it has been concluded that its active site is accessed via a lidded channel [17]. Furthermore, while the enzyme contains two Ca^2+^ ions, only one has a catalytically critical role, while the other helps to provide structural stability [17]. One of the clearly proposed catalytic mechanisms involves a Histidine–Histidine (His-His) catalytic dyad as shown in Figure 1 [18,19]. More specifically, it has been proposed that one histidyl (His115) helps in generation of a reactive hydroxyl by acting as a general base to deprotonate a water molecule. Meanwhile, the second histidyl (His134) acts as a proton shuttle to help with increasing the basicity of His115 [11,20]. Unfortunately, the exact mechanism of PON1 remains unclear. For example, it is known that ethyl acetate is not hydrolyzed while γ-butyrolactone, which contains the same number of atoms and ester moiety, is hydrolyzed by PON1 [1,21].

Significantly, however, human PON1 possesses two common polymorphic sites. One occurs at residue 55 and is normally either a leucine or methionine, and results in an increase at Paraoxonase activity and quantitative differences in enzyme concentration [22]. The other site exists at residue 192 and is either a glutamine (Q) or arginine (R), and as a result are commonly referred to as the Q and R isozymes, respectively. Importantly, the two isozymes (QPON1 and RPON1) have marked qualitative differences [21,23,24,25,26]. As noted above, PON1 possesses hydrolytic activity against organophosphorus compounds which includes some therapeutic drugs, and toxins, such as Paraoxon or chemical warfare agents such as Soman, Sarin and Tabun [13,27,28,29]. However, measurement of the Paraoxonase activity of human serum samples produces a bimodal distribution and is due to the Q/R polymorphism [25]. More specifically, the Q-isozymes exhibit lower activity, while the QR heterozygotes and R homozygotes possess higher activity [8,21,30] against most of the substrates. Consequently, it has been suggested that the Q/R polymorphism of PON1 affects an individual person’s response to, for instance, several toxic substrates [21,31]. Thus, PON1 polymorphism is of considerable interest due to its possible association with a number of diseases such as cardiovascular disease [32,33,34,35,36,37], carotid atherosclerosis [38], Parkinson [39,40], panic disorder [41], multiple sclerosis [42] and Alzheimer’s disease [43]. Previously, PON1 esterase and organophosphatase activities have been described in the literature and included data on substrate structure–activity relationship, lipoprotein effects, kinetics, HDL binding and the effects of salts on activities [24,25,26,27,30,44]. In particular, it has been experimentally reported that the different hydrolyze activities observed for QPON1 versus RPON1 are due to the molecular structure of the substrate [18,21,28]. Unfortunately, despite the physiological importance of PON1 and the clear need to develop better ligand and therapeutic agents, the mechanistic-related explanation for the different reaction rates of QPON1 and RPON1 remains unclear.

In this study, we have complementarily applied multi-scale computational chemistry modeling and QSAR to gain insights into the hydrolysis rate of PON1 and its two isozymes. However, to our knowledge, there has not yet been published any study explaining the different rate of activity of the QPON1 and RPON1, especially by means of quantitative structure–activity relationship (QSAR) approach. Despite the physiological importance, the explanation of different reaction rates for QPON1 and RPON1 has never been completed before.

More specifically, two QSAR models have been built and developed for the activity of the QPON1 and RPON1 against 30 substrates. In addition, unbounded (i.e., no substrate) and substrate-bounded QPON1 and RPON1 have been investigated by systematically applying Docking, Molecular Dynamics (MD) and QM/MM methods to gain an understanding of the effect of Q192R mutation on the active site architecture.

## 2. Materials and Methods

Experimental rates of hydrolysis for QPON1 and RPON1 were reported in the literature by, for example, Billecke et al. [21] and Davies et al. [28]. From the available experimentally studied ligands, a total of 30 compounds (lactones and thiolactones of varying sizes, phenyl acetate and five important organophosphorus compounds) were selected and used for the QSAR studies of QPON1 and RPON1. PON1 is not a Paraoxonase nor an esterase but a lactonase [11], that is why most of the database is composed of lactones. The species selected are shown in Table 1 along with, for convenience, their experimentally measured rates (from ref. [21,28]) of hydrolysis by both QPON1 and RPON1). Phenylacetate is generally accepted as one of the best substrates for experimental studies [45,46,47]. As a result, it was used as the reference compound for experimental studies by, for instance, Billecke et al. [21]. Hence, we have also chosen to relate all of the calculated lactonase and organophosphatase activities to the aryl esterase activity of phenyl acetate.

The Reactant-Complex (RC) model is used in the substrate–enzyme computational models (Docking, MD and QM/MM) (Figure 1). The most reliable and suitable pose was selected from docking results for further MD, QM/MM calculations according to the position and the distance of Ligand, His115 and Ca^2+^ ion.

**Figure 2:**
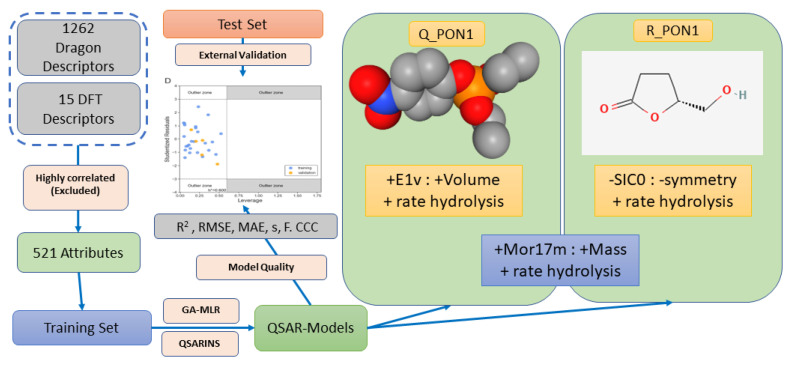
Schematic illustration of the QSAR modelling steps including descriptor generation, training and test sets created and GA-MLR modelling.

The general computational workflow used is illustrated in Figure S1 (Supplementary Materials), while the steps used to create the QSAR models are summarized in Figure 2.

It is noted that we have used similar approaches to develop QSAR models and studies, including analysis for various biological activity and toxicity [48,49].

Genetic algorithm-multi-linear regression (GA-MLR) was performed to develop robust QSAR models. The statistical quality and internal predictivity of the best MLR models were judged by the following statistical parameters: the coefficient of determination (R^2^), the leave-one-out cross-validation (Q^2^_LOO_) and leave-many-out cross-validation (Q^2^_LMO_). In addition, for each model the standard error of estimation was reported. Root Mean Squared Errors (RMSE) for the training (RMSE_TR_) and external prediction sets (RMSE_EXT_), that summarize the overall error of the model, were calculated as an additional measure of the accuracy for the generated QSAR models.

In the case of good external prediction, the calculated values should be close to the observed activity values. The presence of outliers in any model may change its predictive accuracy. Fortunately, there are many methods to detect outliers including identification of those compounds with significantly higher standard residuals from regression-based techniques [50,51,52]. A compound can be identified as a response outlier in MLR models only if the standardized residual is greater than three standard deviation units. In this present study, those chemicals which were structurally very influential in determining the model statistics parameters (i.e., creating leverage effect), were indicated via a Williams plot. To construct the Williams plot, hat values, *hi*, were calculated according to the following equation:

*hi* = X_i_(X^T^ X)^−1^X^T^_i_ (i = 1,………n) (1)

where X_i_ is the descriptor vector of the compound ‘i’; X is the descriptor matrix derived from the training set descriptor values; and X^T^ is its transpose matrix. The critical hat value (h*) was determined as:

h* = 3(*p* + 1)/*n* (2)

where *n* is the number of training compounds and *p* is the number of selected descriptors. Thus, the leverage and the standardized residual were combined for the characterization of the applicability domain (AD). The AD of the models was then verified using the ranges of descriptors that appeared in the model and activity values, and the leverage approach. Compounds in the test set that were predicted due to extrapolation of the model (i.e., fall outside the AD) were detected when their leverage values were greater than the critical hat value (h*) [51,52].

First, the Molecular Operating Environment’s (MOE) [53] MM energy minimization tool was used to allow the substrates to relax and remove any atoms’ interference (Force Field: Amber14: EHT; R-Field 1:80; Cutoff [8,10]) that were then imported into the Dragon 6.0 software [54] to calculate molecular descriptors. A subset of 1276 molecular descriptors were selected from the initial set of more than 4500 calculated descriptors. For the GA-MLR, these Dragon-derived descriptors were then combined with 15 DFT-derived descriptors: total energy (a.u.), RMS (a.u.), dipole moment (debye), zero-point corrected energies, thermal (298.15 K) corrected energies, enthalpy (298.15 K) corrected energies, Gibb’s free energies (298.15 K), HOMO and LUMO energies, hardness and softness, electronegativity, electrophilicity and frequencies and intensities of (P)C=O stretching. It is noted that all compounds in the dataset contain a carbonyl or phosphonyl group (see Table 1), thus, the frequency and intensity of stretching of this groups were included in the descriptors as they are central to the mechanism and possibly the rate of reaction. Hardness, softness, electronegativity and electrophilicity were calculated from HOMO and LUMO energies by standard approaches. Optimized structures and harmonic vibrational frequencies for the dataset compounds were obtained at the M06-2X/6-31G(d,p) level of theory using Gaussian 16 [55]. To perform the analysis, a logarithmic function of experimental rate of reaction values (log(exp)), which were considered as a response variable, and 1291 independent variables were imported into the QSARINS 2.2.1 software [56] to apply a GA–MLR methodology to select the most relevant descriptors. Highly correlated (95%) descriptors (785) were eliminated; finally, 521 descriptors were selected for GA-MLR. The entire data were divided between a training and test set (80:20, respectively) during GA-MLR. Test set compounds were chosen as each fifth by descending order of response. The training and test sets were used to build the QSAR model and for validation, respectively. Finally, the descriptors listed in Table 2 were selected as the most relevant ones for each of QPON1 and RPON1. The density distribution plots of the selected descriptors (violin plots) for both QPON1 and RPON1 models are showed in Figures S5 and S6.

*Docking, MD and QM/MM:* A suitable X-Ray crystal structure of human serum PON1 was obtained from the protein data bank (PDB ID: 3SRG) and used as a template for further treatment. Lys192 was mutated to Arg and Glu amino acids, using PyMol software [57], to obtain a structure for RPON1 and QPON1. The crystal structure preparation and docking were completed using MOE package. The energy minimization results were achieved by using the energy minimization algorithm in MOE, and Amber14 forcefield was used [58]. Moreover, the best pose, from the docking, was selected for further MD calculation. Two different sets of QM/MM calculations were performed; the first one is substrate unbounded, and the second one is enzyme–substrate (selected substrates) complexes (Table 3).

Holoenzyme (i.e., no bound substrate) forms of both QPON1 and RPON1 were modeled to better understand the effects of the mutation on the active site. The topologies for the substrates were created using AmberTools22 [59] using General Amber Forcefield (GAFF) [60]. The enzymes’ topologies were created using Gromacs2021.2 package [61] and Amberff99SB force field [62]. In particular, the enzymes were solvated by a 5 Å spherical layer of water molecules using the SPCE tool in GROMACS 2021.2, which was also used for all MD simulations. Counter ions were added to reach the neutral state. The energy of each structure was then minimized using GROMACS 2021.2 energy minimization algorithm. The final structure was then allowed to thermally relax at a constant temperature and pressure. In particular, velocity rescaling (V-rescale) [63] and Berendsen [64] thermostats were coupled with the equations of motion for the NVT and NPT equilibration steps, where a 2-fs step time was set for numerical equation. More specifically, the systems were equilibrated at 310 K for 1 ns, after which the system was set to 20 ns of MD production. These time periods were selected to better understand the motion nature of the ligands, and the effect of the mutations on the active site, and to allow the water molecules to diffuse inside the active site. The same protocol was used for all systems. Default cut-offs for columbic electrostatic interactions and Van der Waals effects were used, i.e., they decay over distance with a final cut-off of 10 Å. A suitable structure from the MD calculations was then selected and used for further treatment by QM/MM for γ-Butyrolactone, Phenylacetate, Paraoxon and Sarine.

All QM/MM calculations were performed using the ONIOM formalism in Gaussian 16 [55]. Optimized geometries were obtained at the ONIOM(B3LYP/6-31G(d,p):AMBER96) level of theory. AMBER96 is the default forcefield in Gaussian 16. The high layer (QM layer) included the active site: the selected substrates, the calcium ion, crucial residues, and waters within 4.5 Å of it. The remaining region (MM-layer), which included the residue 192, was described using the AMBER96 force field.

## 3. Results and Discussion

As noted above, PON1 is a multi-functional antioxidant enzyme that can hydrolyze organophosphorus compounds, aryl and vinyl esters, lactones and thiolactones [45]. Thus, in order to create an inclusive QSAR model [62,63,64,65], each of these functional groups were included in the dataset (see Table 1).

**Figure 3:**
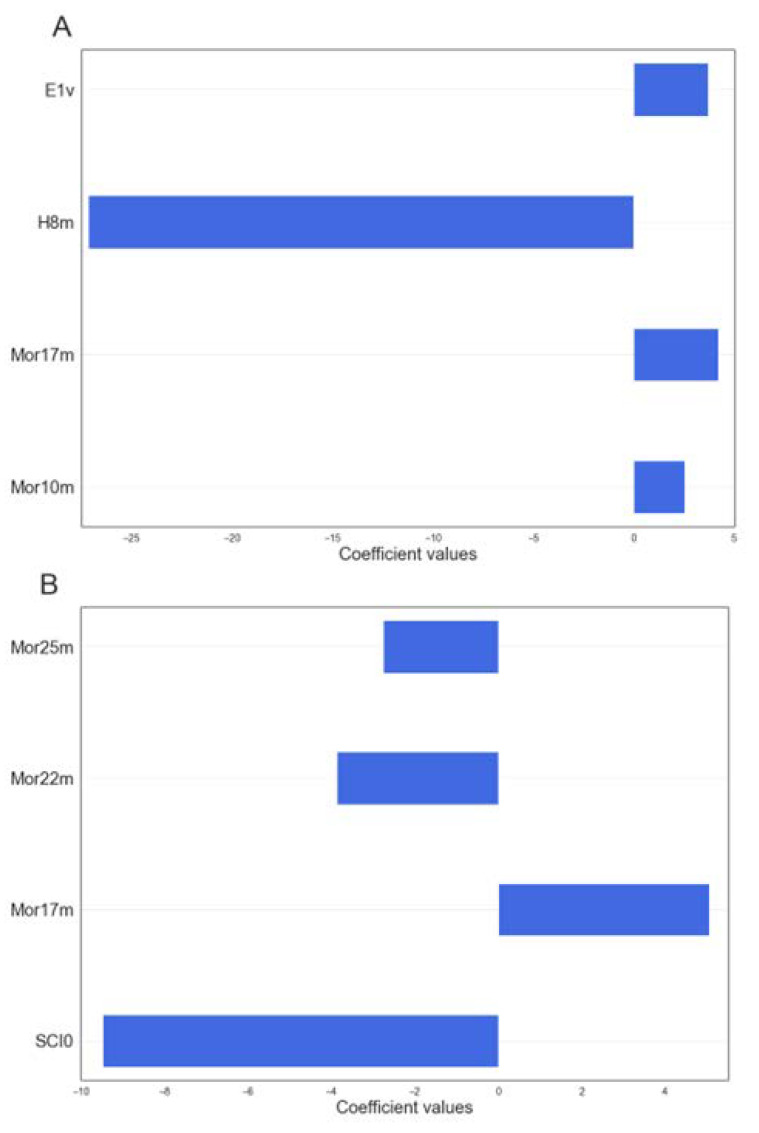
The relation between the descriptors and the rate of hydrolysis for (**A**) QPON1 by Equation (3) and (**B**) RPON1 by Equation (4).

A best model for QPON1 and RPON1 was selected using the multi-criteria decision making (MCDM) tool within QSARINS, and the resulting equations are shown below; Equations (3) and (4), respectively. Meanwhile, the relation between the descriptors and the rate of hydrolysis of the *Q* and *R* isoenzymes is summarized in Figure 3. As can be seen, the responses are a logarithmic function of rate of hydrolysis and its distribution across the dataset are depicted in Figures S7 and S8. According to the selected models in Equations (3) and (4), *Mor10m*, *Mor17m* and *E1v* are directly proportional to the *QPON1* activity rate and H8m shows a negative effect in the response. For *RPON1* (Equation (4)) the only descriptor with a positive contribution to the activity is *Mor17m* (Figure 3). It should be noted that *Mor17m* appears in both models and with positive contributions in both cases. Besides the density distribution plots of the descriptors (violin plots) for both *QPON1* and *RPON1* models are shown in Figures S5 and S6.

Log (A_QPON1) = −0.487 + 2.066*Mor10m + 3.970*Mor17m + 3.319*E1v − 25.907*H8m (3)

Log (A_RPON1) = 4.879 − 9.472*SIC0 + 5.054*Mor17m − 3.861*Mor22m − 2.763Mor25m (4)

As summarized in Figure 3, there are 3D-MoRSE descriptors in both models (two for Q model and three for R model). The 3D-MoRSE indicates the 3D molecular representations of structure-based electron diffraction; additionally, these descriptors are member of Highly Conformational Dependent (HCD) descriptors [66,67]. All four 3D-MoRSE descriptors are weighted by mass, which practically eliminates the role of hydrogen atoms, while significantly increases the effect of phosphorus, sulfur and chlorine and greatly increases the effect of heavy atoms such as bromine and iodine on the values of 3D-MoRSE descriptors [66]. Moreover, in the case of the model for the Q isoyenzyme, we have the E1v molecular descriptor that is a WHIM index weighted by Van der Waals volume, indicating some role related to the conformation of the molecule. So, it can be concluded that the conformation and heavy atom profile of the substrates are very important factors for the rate of reaction, especially for RPON1.

**Figure 4:**
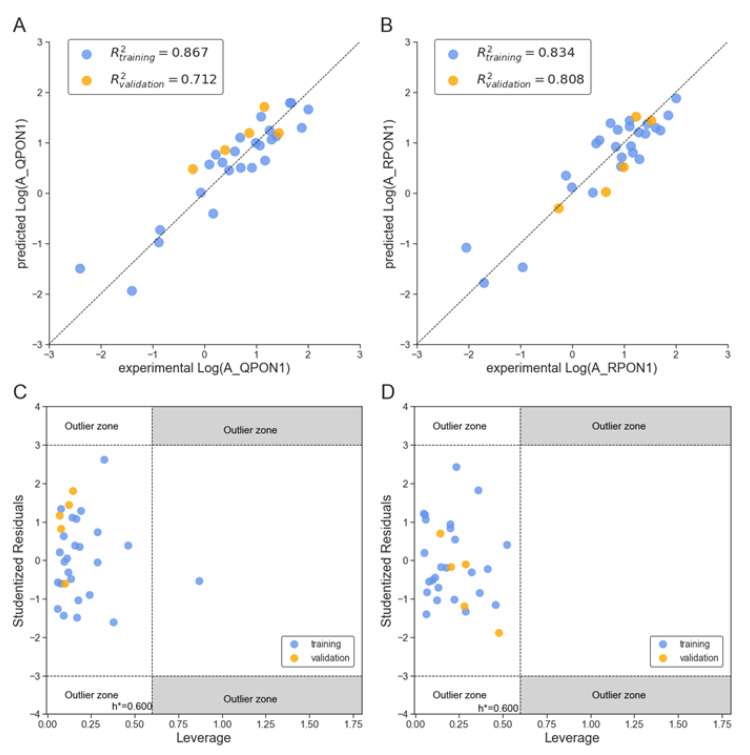
Plots of the experimental endpoint vs. predicted for (**A**) QPON1 by Equation (3) and (**B**) RPON1 by Equation (4), and the corresponding Williams plots for (**C**) QPON1 and (**D**) RPON1. Molecules in the training set are indicated by blue dots, while those in the validation set are indicated by orange dots.

We also compared the experimental endpoint vs. predicted values using the current models (Equations (3) and (4)), as well as Williams plots, and shown in Figure 4. For both QPON1 (Figure 4A,C) and RPON1 (Figure 4B,D). there is generally a good agreement between experimentally obtained and predicted data. The fitting criteria, external and internal validation results are also supporting the agreement (Table 4 and Table 5). The *y*-scrambling results for both models (QPON1 and RPON1) are provided in Figure S4.

As noted in the introduction, experimentally it has been observed that QPON1 exhibits a higher rate of hydrolysis for some substrates (e.g., diazoxon), while RPON1 exhibits a higher rate of hydrolysis for others (e.g., paraoxon) [6,21,37]. Thus, we then considered a Molecular Dynamics (MD) simulation to examine the interaction between the substrates and the active site vs. time. Moreover, the MD calculation will also give an insight into the effect of the mutants, QPON1 and RPON1, on the active site’s residues. The MD calculation was completed for four different substrates from different substrate classes, i.e., γ-Butyrolactone, Phenylacetate, Paraoxon and Sarine (Table 3). The distances between Hist115 and the selected substrates, Hist115 and the Ca^2+^, and the Ca^2+^ and the residue 192 are critical for the mechanism and rate of reaction, for this reason they are graphically (Figure S4) and averagely (Table 6) reported. Additionally, the Root Mean Square Deviation (RMSD) of the backbone, the Gln192/Arg192 and the substrates were obtained based on the enzyme–substrate complex (RC) (Figure 1) are reported in Figure S3.

**Figure 5:**
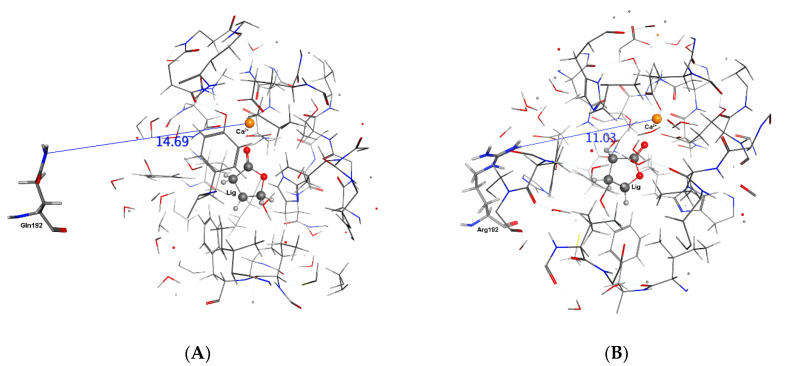
The distance between Glu192/Arg192 and Ca^2+^ in the γ-Butyrolactone complexes after 20 ns. (**A**) QPON1. (**B**) RPON1.

**Figure 6:**
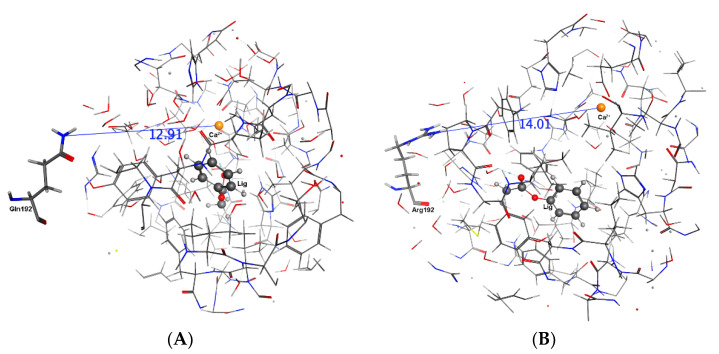
The distance between residue192 and Ca^2+^ ion in the active site of the Phenylacetate complex after 20 ns. (**A**) is the QPON1. (**B**) is the RPON1.

Experimentally, the rate of hydrolysis of phenylacetate for both QPON1 and RPON1 is set as 100 with relative rates for each isozyme then determined with respect to these values. The MD calculations may support these results. In complex **1**–**4**, the average distances between Arg/Gln192---Ca^2+^, His115---Ca^2+^ in QPON1, and RPON1 isoenzymes over 20 ns are mentioned in Table 6. The distances are relatively smaller in RPON1 than that of the QPON1 in all selected complexes except sarin. Thus, the closer the distance between mutation site residue 192 to the active site is, this causes a shortening of the distance between the His115 and Ca^2+^ ion. This phenomenon is reversed when sarin is a ligand, which may be related to the different orientation of sarin than others in the active site (Figure 5). Interestingly, in the Phenylacetate complex (Figure 6), the distance between Gln192---Ca^2+^ is almost as the same as Arg192---Ca^2+^ which means the mutation is not effecting the distance to the active site, yet the distance between His115---Ca^2+^ is still smaller in the RPON1 Isoenzyme than QPON1 Isoenzyme.

**Figure 7:**
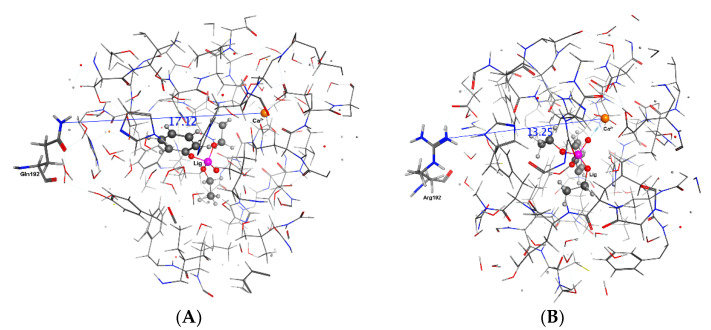
The distance between Glu192/Arg192 and Ca^2+^ ion in the active site in the Paraoxon system after 20 ns. (**A**) is the QPON1. (**B**) is the RPON1.

**Figure 8:**
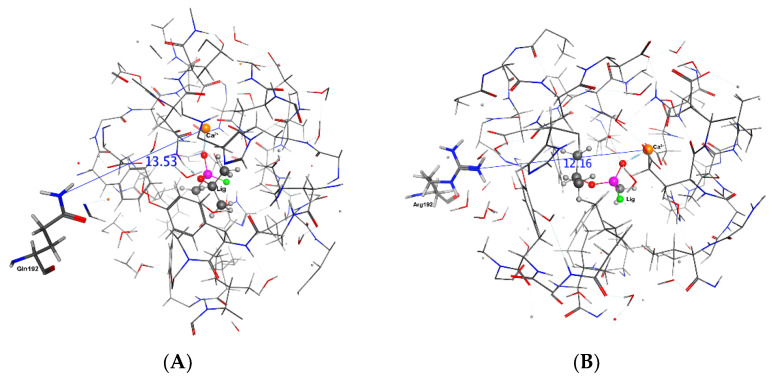
The distance between Glu192/Arg192 and Ca^2+^ ion in the active site in the Sarine–Enzyme complex after 20 ns. (**A**) QPON1. (**B**) RPON1.

It can be seen in Table 6 that including a ligand in active site is not causing the same effect on reported distances. For example, penetration of sarin (Figure 8) to the active site is increasing the distance between the His115 and Ca^2+^ ion for both isozymes; however, paraoxon (Figure 7) and phenylacetate are reducing for R and increasing for Q. It can be concluded that the mechanism is highly sensitive to mutation and substrate molecular structure and this conclusion is in harmony with QSAR and experimental literature [11]. However, there is a unique substrate effect on the distances between the mutated residue and Ca^2+^. All substrates increased the distance between the mutated residue and Ca^2+^ at QPON1 and reduced at RPON1, except Paraoxon (Table 6, Figure 6, Figure 7 and Figure 8).

**Figure 9:**
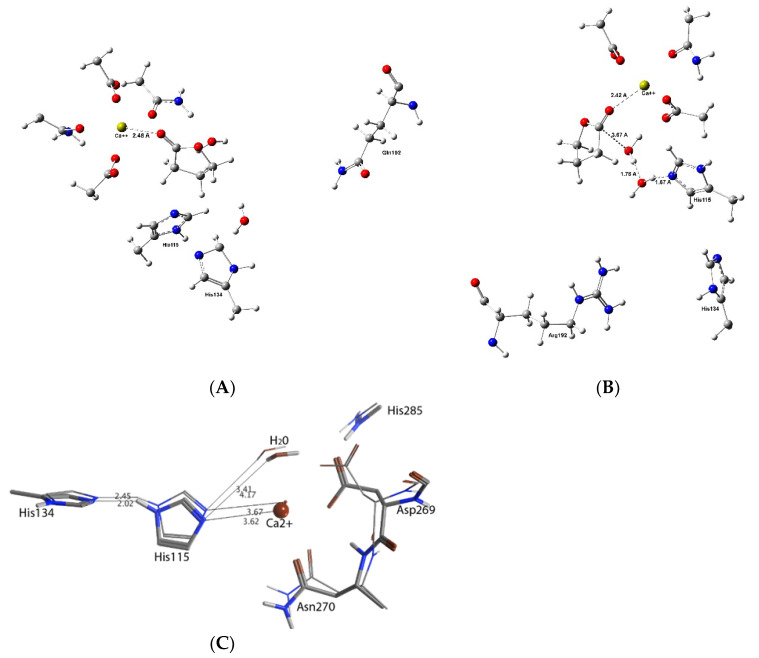
the QM/MM results of the QPON1 and RPON1 of the γ-Butyrolactone system. (**A**) is the QPON1, (**B**) is the RPON1, (**C**) superimposition of the optimized structures of selected key active site residues, the Ca^2+^ ion and mechanistic water for QPON1 and RPON1.

As described in the introduction, the PON1 catalyzed hydrolysis mechanism has been studied experimentally and computationally (using a QM/MM approach [19]) (see Figure 1). It was concluded that His115 plays a key role in, for example, activating the mechanistically important water molecule. In fact, activation of the water molecule was identified as the rate-limiting step in the overall mechanism [18,19]. The above results obtained using MD suggest that the active sites of the isozymes are in a dynamic motion, and they are affected by the mutation even in a substrate unbound state (Figure 9C). Thus, using QM/MM, in which the QM-region was described using DFT methods (see Computational Methods), the structures of the active sites of both QPON1 and RPON1 with γ-butyrolactone were examined. That is, the question of what impacts does the Q192R mutation have on their native (no substrate) active sites was asked. The optimized positions of selected key active site residues, the Ca^2+^ ion and mechanistic water for QPON1 and RPON1 were then further examined to give an insight into the difference that the two mutants brought to the active site.

Figure 9 is representing the QM/MM results; the results are quite interesting. First of all, the ligand-free active site was optimized using Quantum Mechanics Techniques. It shows that the Q192R mutation affects the active site’s geometry despite being in the third shell away from the active site, QM region (Figure 9C). Penetration of γ-butyrolactone is massively changing the active site architecture in different magnitudes. For both of the isozymes, one more water molecule is accompanying the substrate, but their orientation is completely different. In R-PON1, both of the water molecules are in an active role and the His115-H_2_O-H_2_O-Substrate-Ca^2+^complex is looking ready to initiate a chemical reaction (Figure 9B). There is no such orientation in Q-PON1, the water molecules are not located between Hys115 and substrate to trigger a reaction (Figure 9A). Not surprisingly, the experimental data are suggesting a four-fold faster hydrolyze for R-PON1. This conclusion is in a harmony with QSAR results which reflects a very important role for substrate 3D-geometry on the rate of reaction. The distance between His115 and water is 3.41 and 4.17 A in R and Q isoenzymes in substrate-free models, respectively, and, in our understanding, the shorter distance between histidine and water may permit a more organized geometry when the substrate and extra water penetrates the active site.

## 4. Conclusions

In this study, using a computational QSAR approach, the structure–activity model was developed to examine the properties of the substrates of Paraxonase-1 (PON1), in order to help provide insights into substrate properties that may influence the relative rates of reaction (hydrolysis) in the Q- and R-isozymes of PON1. In addition, we have also complementarily used Protein–Ligand Docking, Molecular Dynamics (MD) and QM/MM approaches to examine the effect of the Q versus R mutation at position 192 of PON1 on binding of selected substrates in the active sites, and possible differences in the active site structure.

In addition to analyzed main statistical performance values in the QSAR component of this study, the applicability domain of the models was also analyzed by applying the Williams plot technique. The selected significant descriptors and obtained model performance results revealed the main factors that influence the activity of the PON1 isozymes. For QPON1, it was found that the substrate properties that were most likely to be important for the interactions included molecular mass weighted 3D-MoRSE descriptors (Mor10m, Mor17m) and a Van der Waals-weighted WHIM descriptor (E1v). That is, for QPON1, the most important descriptors of the substrate are related to their three-dimensional molecular structure-dependent conformation. In the case of RPON1, three mass weighted molecular descriptors were included in the QSAR model (Mor17m, Mor22m, Mor25m), all of them in the same type of descriptor family (3D-MoRSE). For instance, it is important to notice that both of the QSAR models share one descriptor in common Mor17m having in both cases a positive effect on the hydrolysis rate for QPON1 and RPON1.

As noted, Protein–Ligand Docking, MD and QM/MM methods were applied to obtain greater insights into the effects of the residue 192 mutation, specifically Q- vs. RPON1, on substrate binding and active site structure as the Q and R polymorphisms have been experimentally observed to hydrolyze almost all substrates at different rates. The present computational results suggest that the Q192R mutation does cause structural changes in the active site that include the positioning of the key Ca^2+^ ion and the required mechanistic water. Additionally, penetration of the substrate is causing an extra water molecule in the active site which has a mechanistic role in the RPON1/γ-butyrolactone complex. The discussion on the primary role of the PON1 enzyme is still ongoing [11] because PON1 hydrolyzes a broad range of compounds (aryl esters, lactones, organophosphorus compounds, etc.). According to the produced QSAR, MD and QMMM results, the PON1 active site is very sensitive to Q192R mutation (especially when substrate bound) and substrate 3D-geometry. Experimental results are also referring to such a kind of sensitivity; small changes on the substrate molecular structure cause big differences on the rate of reaction (a more than 30-fold difference between the rate of reaction of γ-butyrolactone and δ-valerolactone for QPON1, eight-fold for RPON1). As a result of this sensitivity, different mechanisms may exist according to isozyme or substrate. To the best of our knowledge, the mechanism suggested in this study for RPON1- γ-Butyrolactone system (Figure 8B) has not been reported before.

## Figures and Tables

**Table 1:** The substrates used in the present study and their experimentally measured rate of hydrolysis [21,28] by QPON1 and RPON1 at [substrate] = 1mM.

No	Structure	Name	Human Q192 PON1 Rate of Hydrolysis	Human R192 PON1 Rate of Hydrolysis	Reference
1	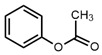	Phenyl acetate	100	100	[21]
2	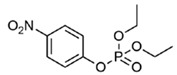	Paraoxon	0.13	0.99	[21,28]
3	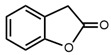	2-Coumaranone	18.3	13.5	[21]
4	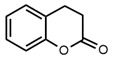	Dihydrocoumarin	14.3 *	17.0 *	[21]
5	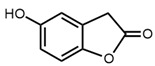	Homogentisic acid lactone	44.0	49.7	[21]
6	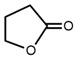	γ-Butyrolactone	2.46 *	9.05	[21]
7	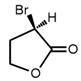	α-Bromo- γ -butyrolactone	47.2	40.8	[21]
8	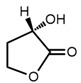	*S*-α-Hydroxy-γ-butyrolactone	8.14	19.6	[21]
9	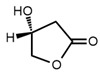	*S*-β-Hydroxy-γ-butyrolactone	0.60 *	0.76	[21]
10	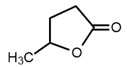	γ-Valerolactone	7.28 *	6.97	[21]
11	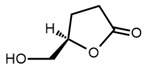	*R*-Dihydro-5- hydroxymethyl)-2(3H)-furanone	1.23	3.29	[21]
12	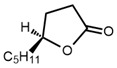	γ-Decanolactone	12.4	19.0	[21]
13	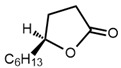	Undecano-γ-lactone	11.8	12.7	[21]
14	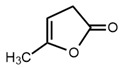	α-Angelicolactone	19.9	14.8	[21]
15	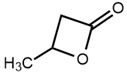	β-Butyrolactone	3.83	7.53	[21]
16	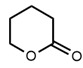	δ-Valerolactone	75.4	71.0	[21]
17	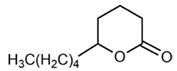	δ-Decanolactone	23.8	28.2	[21]
18	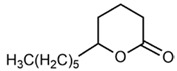	Undecanoic-δ-lactone	27.5 *	32.8 *	[21]
19	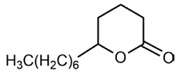	δ-Dodecanolactone	9.65	12.8	[21]
20	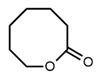	ε-Caprolactone	14.8	25.7	[21]
21	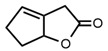	Oxabicyclooctenone	1.67	2.92	[21]
22	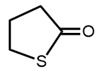	γ-Thiobutyrolactone	0.04	0.11	[21]
23	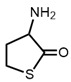	HTL; homocysteine thiolactone	0.004	0.009	[21]
24	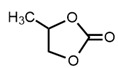	Propylene carbonate	5.02 *	8.80	[21]
25	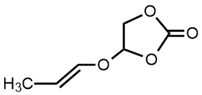	4-(1-Propenyloxymethyl)-1,3- dioxolan-2-one	2.23	2.52	[21]
26	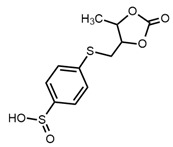	4-[(5-methyl2-oxo-1,3-dioxol-4-yl)methylthio] benzenesulfonate	1.46	9.65 *	[21]
27	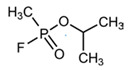	Sarin	0.14	0.02	[28]
28	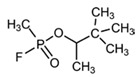	Soman	0.85	0.55 *	[28]
29	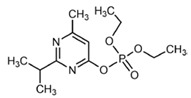	Diazoxon	4.88	4.45 *	[28]
30	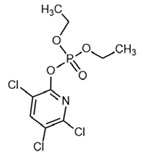	Chlorpyrifos oxon	2.97	5.40	[28]

* Test set compounds.

**Table 2:** Selected molecular descriptors by GA-MLR technique involved in the best models for QPON1 and RPON1.

QPON1		RPON1	
Abbreviation	Descriptor	Abbreviation	Descriptor
Mor10m	signal 10/weighted by mass 3D-MoRSE descriptors	SIC0	Spectral moment 05 from edge adj. matrix weighted by dipole moments
Mor17m	signal 17/weighted by mass 3D-MoRSE descriptors	Mor17m	signal 17/weighted by mass 3D-MoRSE descriptors
E1v	First component accessibility directional WHIM index/weighted by van der Waals volume	Mor22m	signal 22/weighted by mass 3D-MoRSE descriptors
H8m	H autocorrelation of lag 8/weighted by mass	Mor25m	signal 25/weighted by mass 3D-MoRSE descriptors

**Table 3:** Selected substrates and nomenclature of enzyme substrate complexes.

Compound	QPON1 Isoenzyme	RPON1 Isoenzyme
γ-Butyrolactone	Complex1Q	Complex1R
Phenylacetate	Complex2Q	Complex2R
Paraoxon	Complex3Q	Complex3R
Sarine gas	Complex4Q	Complex4R

**Table 4:** Fitting, internal validation, and external validation criteria [67] for Q isozyme model.

**Fitting criteria**
R^2^ tr = 0.867, R^2^adj = 0.841, RMSE = 0.378 MAE tr = 0.305,
CCC tr = 0.929, s = 0.422, F = 32.613
**Internal validation criteria**
Q^2^LOO = 0.766, RMSE cv = 0.501, MAE cv = 0.401,
Q^2^LMO = 0.712, CCC cv = 0.881
**External validation criteria**
R^2^ext. = 0.712, RMSE ext. = 0.491, MAE ext. = 0.463, CCC ext. = 0.628

**Table 5:** Fitting, internal validation, and external validation criteria for R isozyme model.

**Fitting criteria**
R^2^ tr = 0.834, R^2^adj = 0.080, RMSE = 0.407 MAE tr = 0.346,
CCC tr = 0.909, s = 0.455, F = 25.081
**Internal validation criteria**
Q^2^lLOO = 0.726, RMSE cv = 0.522, MAE cv = 0.439,
Q^2^LMO = 0.683, CCC cv = 0.854
**External validation criteria**
R^2^ext. = 0.808, RMSE ext. = 0.372, MAE ext. = 0.297, CCC ext. = 0.854

**Table 6:** The average distances (A) between Arg/Gln192---Ca^2+^, His115---Ca^2+^ in QPON1, and RPON1 Isoenzymes over 20 ns.

Compound	Arg192---Ca^2+^ Distance	Gln192---Ca^2+^ Distance	His115---Ca^2+^ Distance RPON1	His115---Ca^2+^ Distance QPON1
γ-Butyrolactone (Figure 5)	1.107	1.362	0.525	0.944
Phenylacetate (Figure 6)	1.417	1.404	0.468	0.865
Paraoxon (Figure 7)	1.226	1.669	0.233	0.879
Sarine gas (Figure 8)	1.457	1.253	0.921	0.846
Substrate-free	0.970	1.431	0.541	0.688

## Data Availability

Not applicable.

## Supplementary Materials

The following supporting information can be downloaded at: https://www.mdpi.com/article/10.3390/molecules27206780/s1, Figure S1: Schematic illustration of the enzyme–substrate complex modeling strategy used in this study; Figure S2: Schematic illustration of the QSAR modelling steps including descriptor generation, training and test sets created and GA-MLR modelling; MD Graphs. Figure S3A–C: Substrate-free QPON1 and RPON1 MD results including distances between His115-Ca ion, Gln192/Arg192-Ca ion, Gln192/Arg192 RMSD, and the Backbone RMSD. Figure S4: Y-scrambling test resulting graphs for QPON1 model, Equation (3) (A), and RPON1 model, Equation (4) (B); Figure S5: Density distribution of the descriptors values for each variable in a violin plot for the QPON1 model. Figure S6: Density distribution of the descriptors values for each variable in a violin plot for the RPON1 model; Figure S7: Histogram distribution for QPON1 model based on response variable (rate of reaction); Figure S8: Histogram distribution for RPON1 model based on response variable (rate of reaction).
