# Supplementary material for: The Hydrolysis Rate of Paraoxonase-1 Q and R Isoenzymes: An In Silico Study Based on In Vitro Data
Source: Molecules. 2022 Oct 11;27(20):6780. doi: 10.3390/molecules27206780 (PMC9607273; doi:10.3390/molecules27206780)
Supplement: Supplementary file 1 [file molecules-27-06780-s001.zip › molecules-1925606-supplementary.pdf]

The Hydrolysis Rate of Paraxonase-1 Q vs R Isoenzymes: An *in silico* Study Based on *in vitro* Data

Table of Contents

**Figure S1.** Schematic illustration of the enzyme-substrate complex modeling strategy used in this study…………………………………………………………..	**Page S2**
**Figure S2** Schematic illustration of the QSAR modelling steps including descriptor generation, training and test sets created and GA-MLR modelling….	**Page S2**
**Figure S3A–C** Substrate-free QPON1 and RPON1 MD results including distances between His115-Ca ion, Gln192/Arg192-Ca ion, Gln192/Arg192 RMSD, and the Backbone RMSD. ……………………………………………..	**Page S3-S20**
**Figure S4.** Y-scrambling test resulting graphs for QPON1 model, eq. 3 (A), and RPON1 model, eq. 4 (B)……………………….………………………………..	**Page S20**
**Figure S5.** Density distribution of the descriptors values for each variable in a violin plot for the QPON1 model.…………………….………………………	**Page S21**
**Figure S6.** Density distribution of the descriptors values for each variable in a violin plot for the RPON1 model.………………….……………………	**Page S21**
**Figure S7.** Histogram distribution for QPON1 model based on response variable (rate of reaction).……………………….………………………………	**Page S22**
**Figure S8.** Histogram distribution for RPON1 model based on response variable (rate of reaction).……………………….………………………………..	**Page S22**

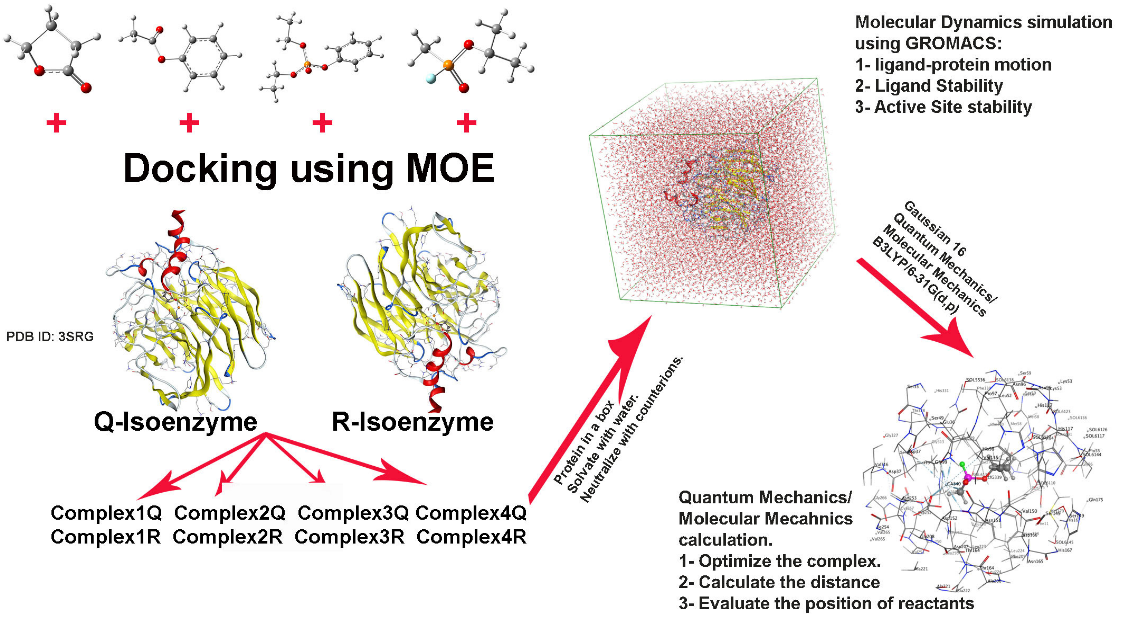

Figure S1. Schematic illustration of the enzyme-substrate complex modeling strategy used in this study.

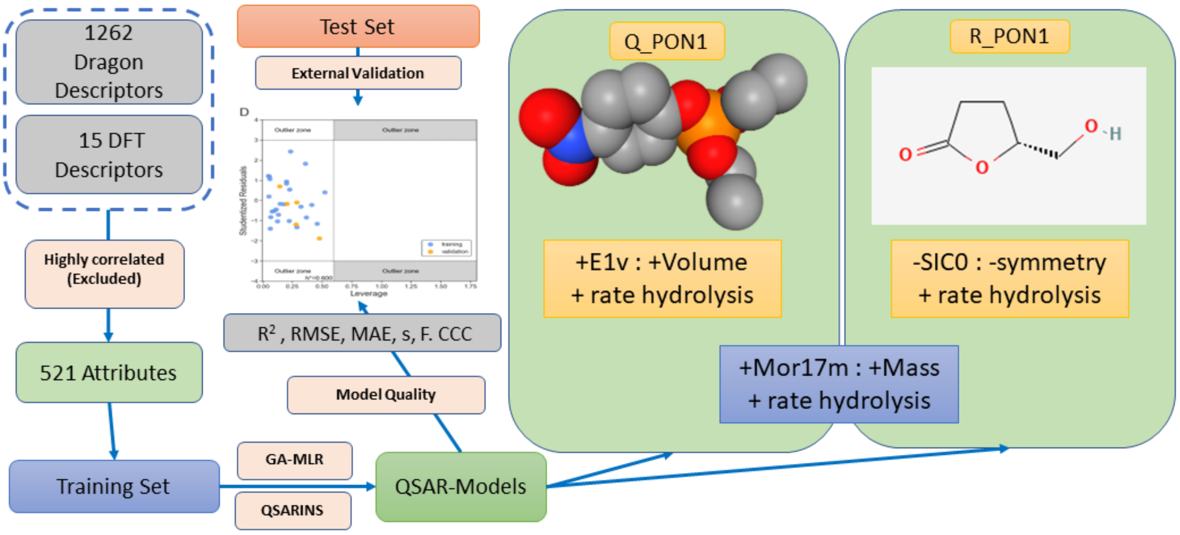

Figure S2. Schematic illustration of the QSAR modelling steps including descriptor generation, training and test sets created and GA-MLR modelling.

Figure S3A. Backbone RMSD, Gln192/Arg192 RMSD, and Ligands RMSD from MD results of the four complexes, γ-Butyrolactone, Phenylacetate, Paraoxon, Sarine gas, and substrate free complexes, including.

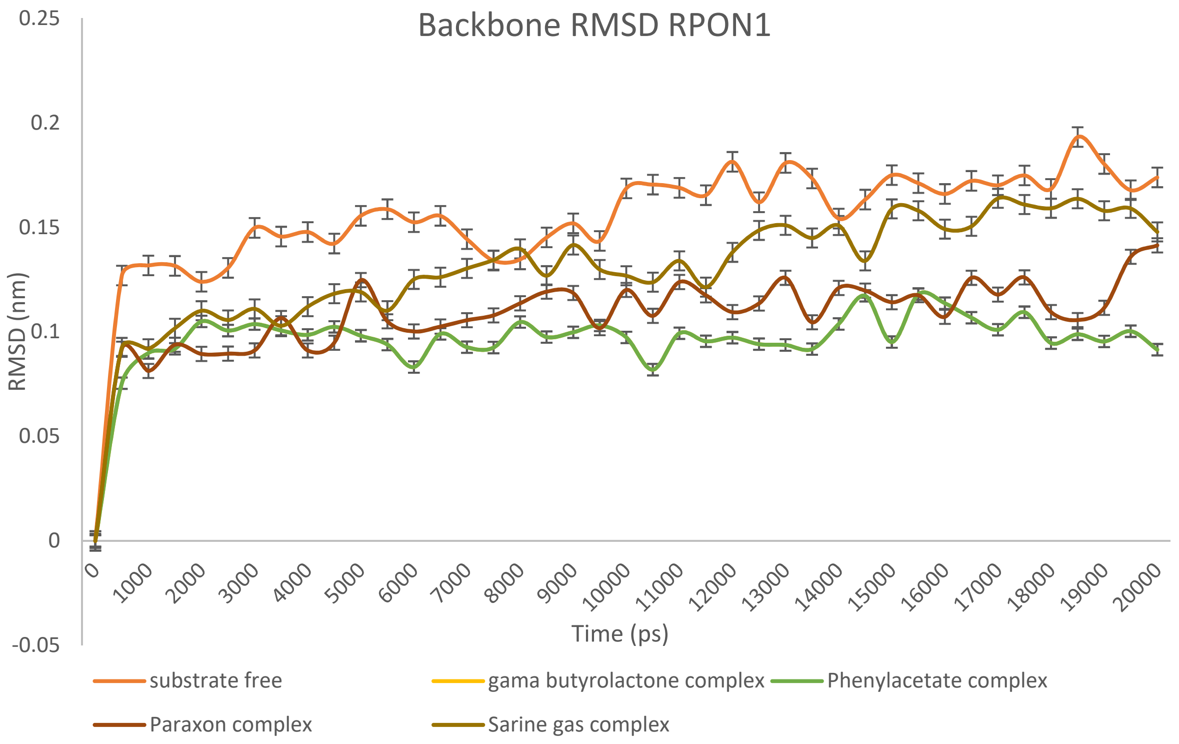

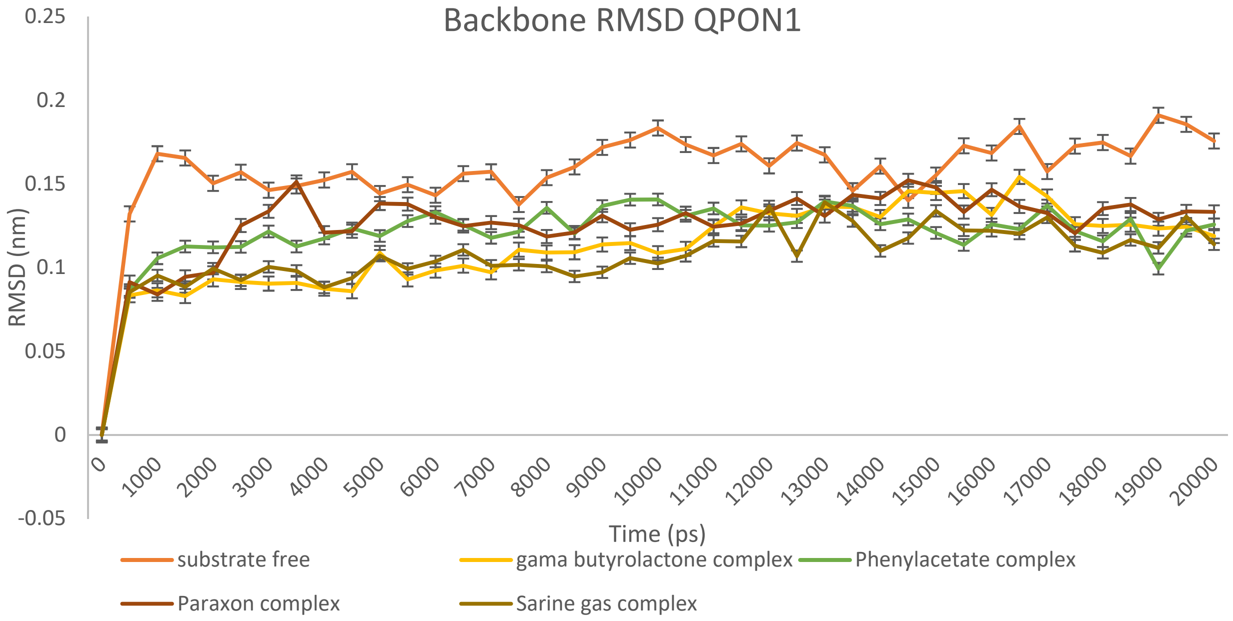

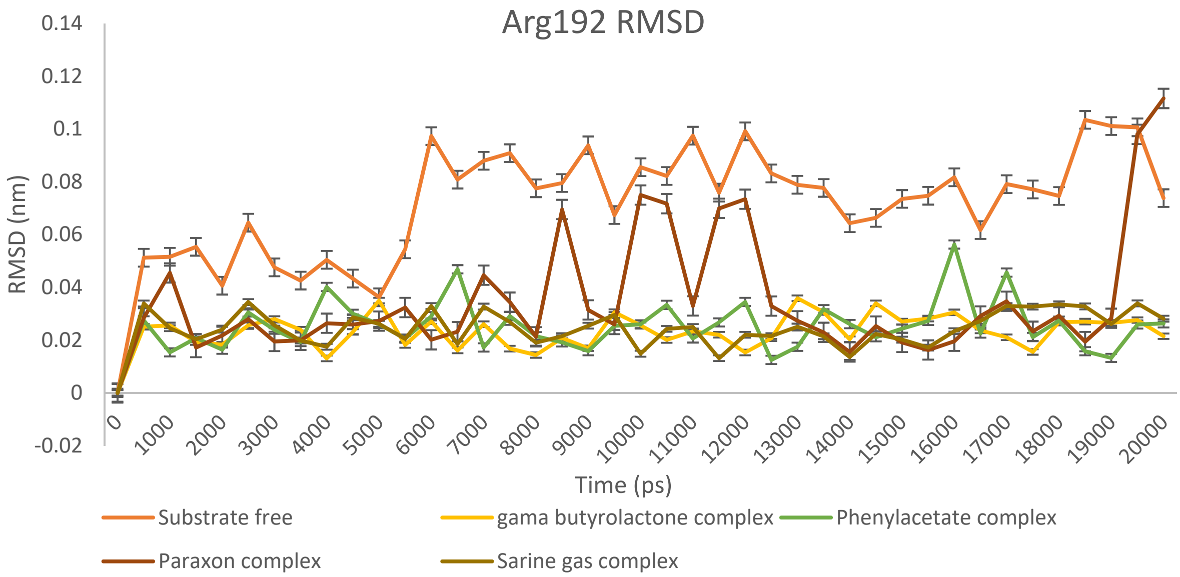
Arg192 RMSD
RMSD (nm)
Time (ps)
Substrate free
gama butyrolactone complex
Phenylacetate complex
Paraxon complex
Sarine gas complex

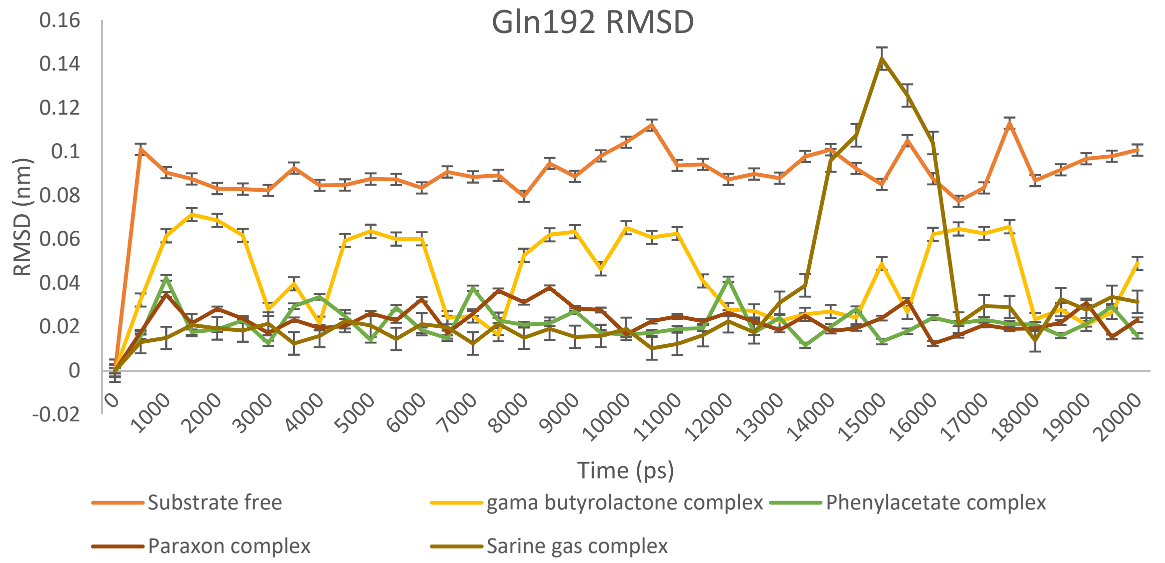
Gln192 RMSD
RMSD (nm)
Time (ps)
Substrate free
gama butyrolactone complex
Phenylacetate complex
Paraxon complex
Sarine gas complex

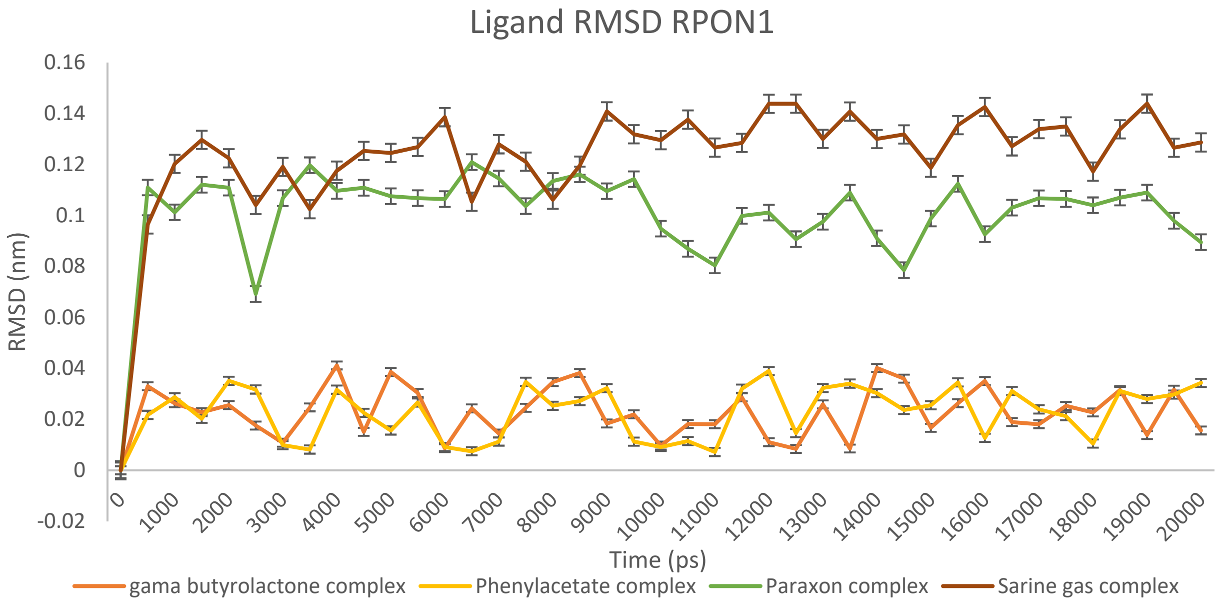

Ligand RMSD RPON1
RMSD (nm)
Time (ps)
gama butyrolactone complex
Phenylacetate complex
Paraxon complex
Sarine gas complex

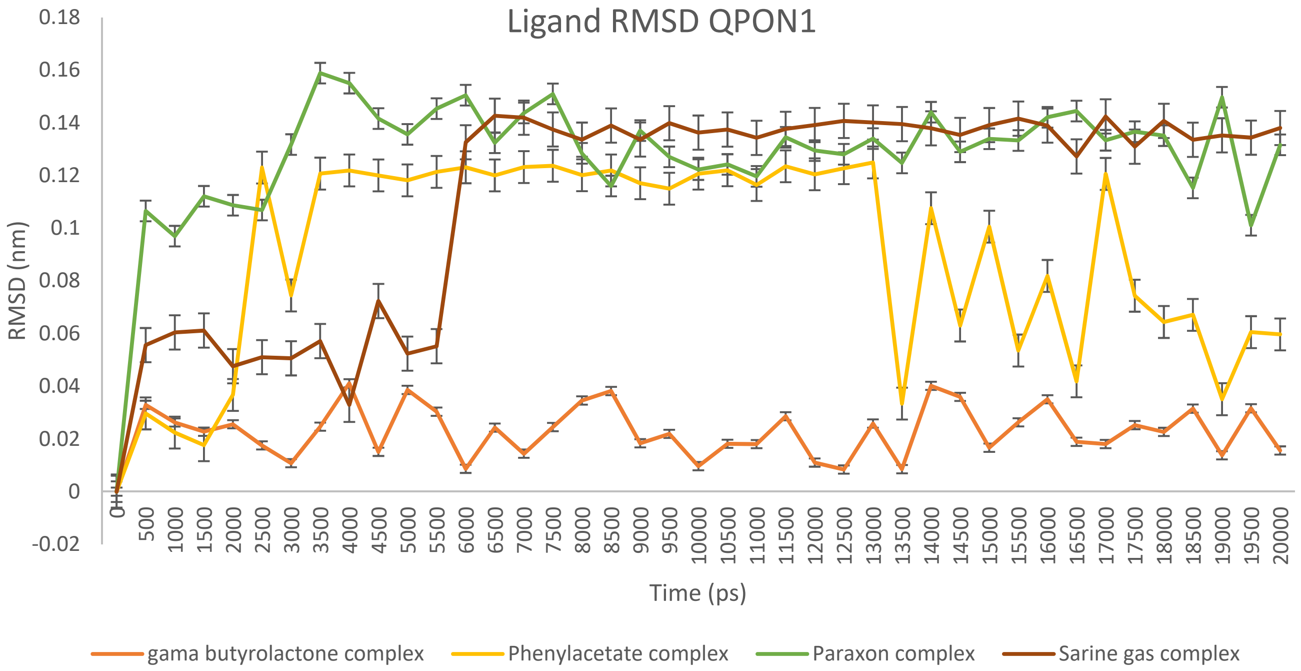

Ligand RMSD QPON1
RMSD (nm)
Time (ps)
gama butyrolactone complex
Phenylacetate complex
Paraxon complex
Sarine gas complex

Figure S3B. Gln192/Arg192-Ca^{2+} ion distance from MD results of the four complexes, γ-Butyrolactone, Phenylacetate, Paraoxon, Sarine gas, and substrate free complexes.

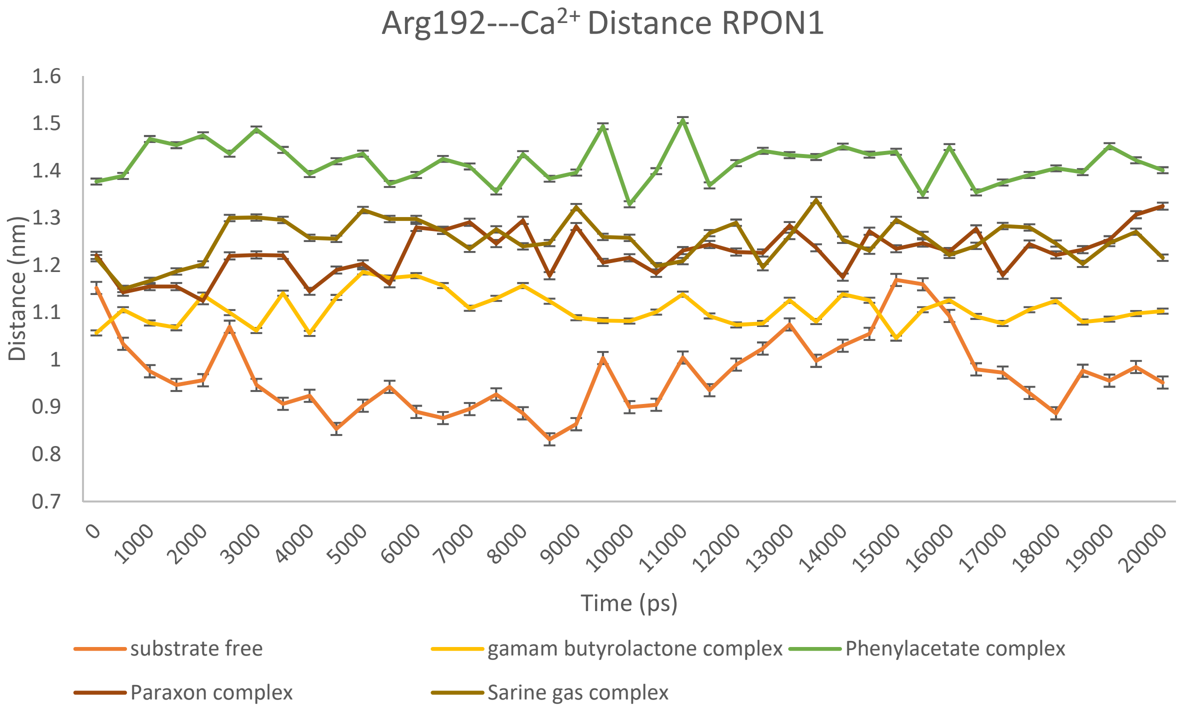

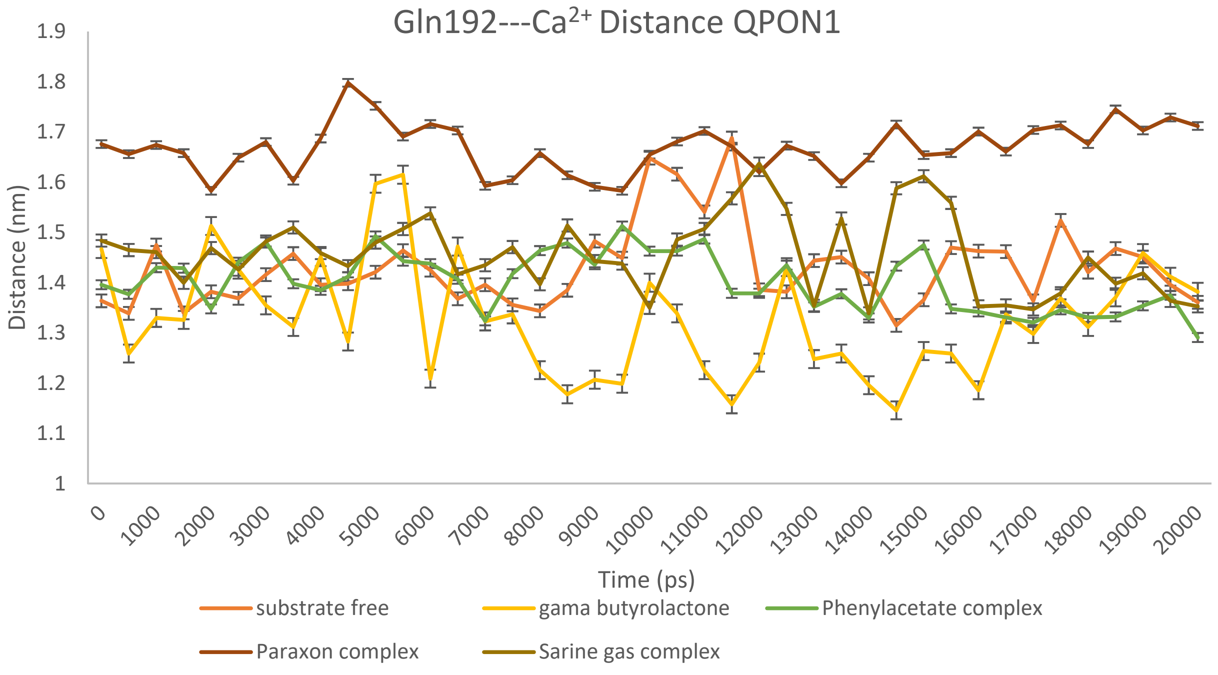

Figure S3C. His115-Ca^{2+} ion distance from MD results of the four complexes, γ-Butyrolactone, Phenylacetate, Paraoxon, Sarine gas, and substrate free complexes.

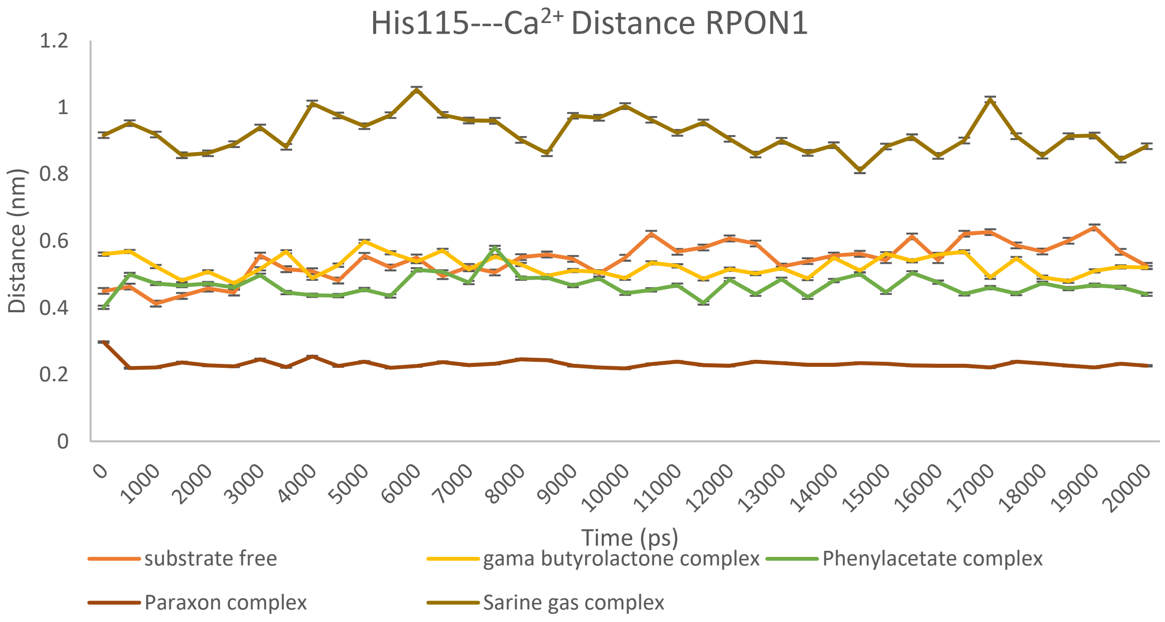

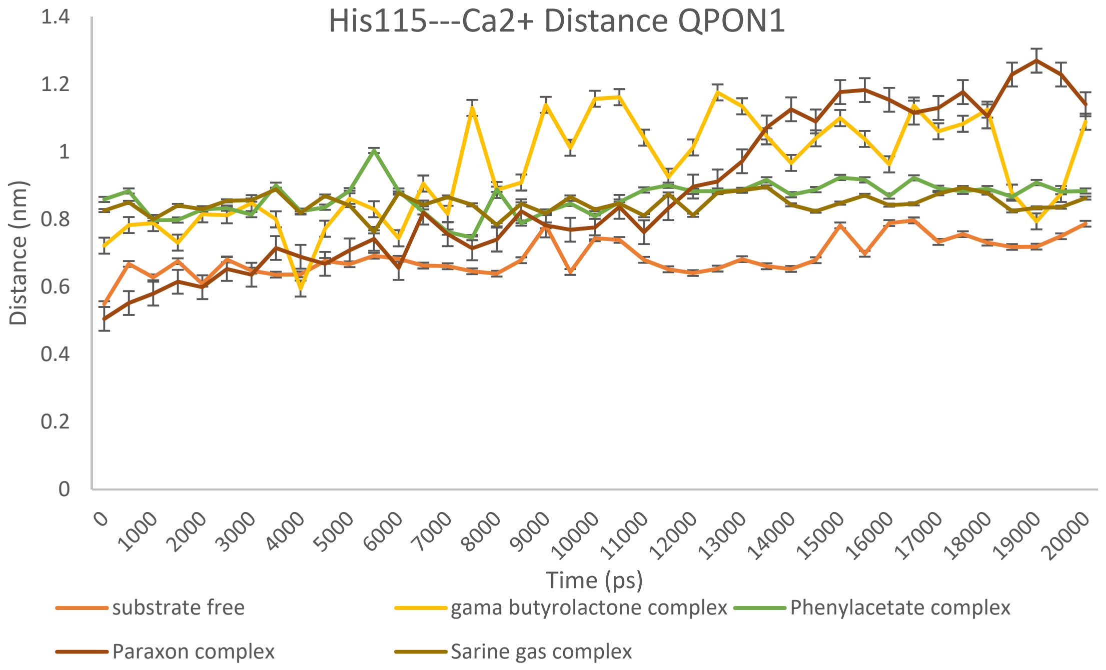

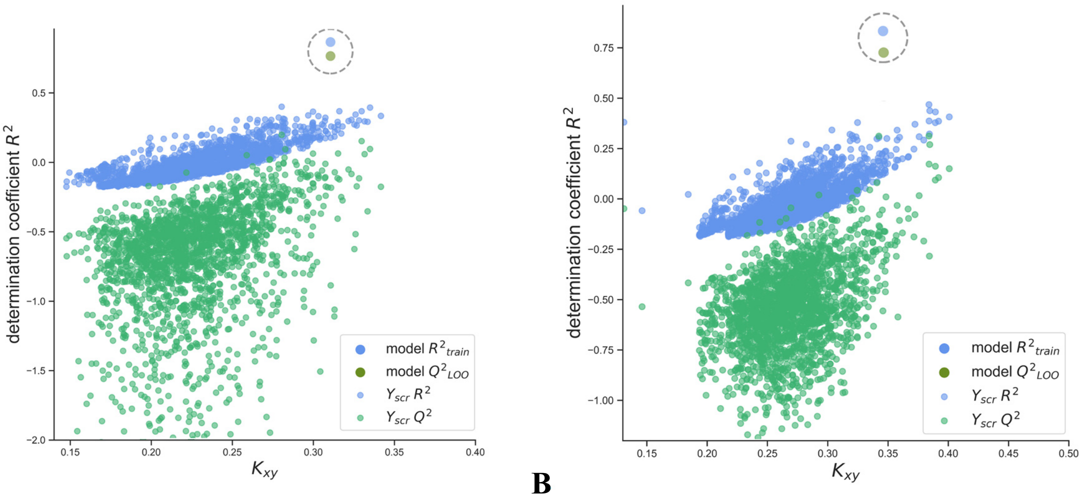

A **B**

Figure S4. Y-scrambling test resulting graphs for QPON1 model, eq. 3 (A), and RPON1 model, eq. 4 (B)

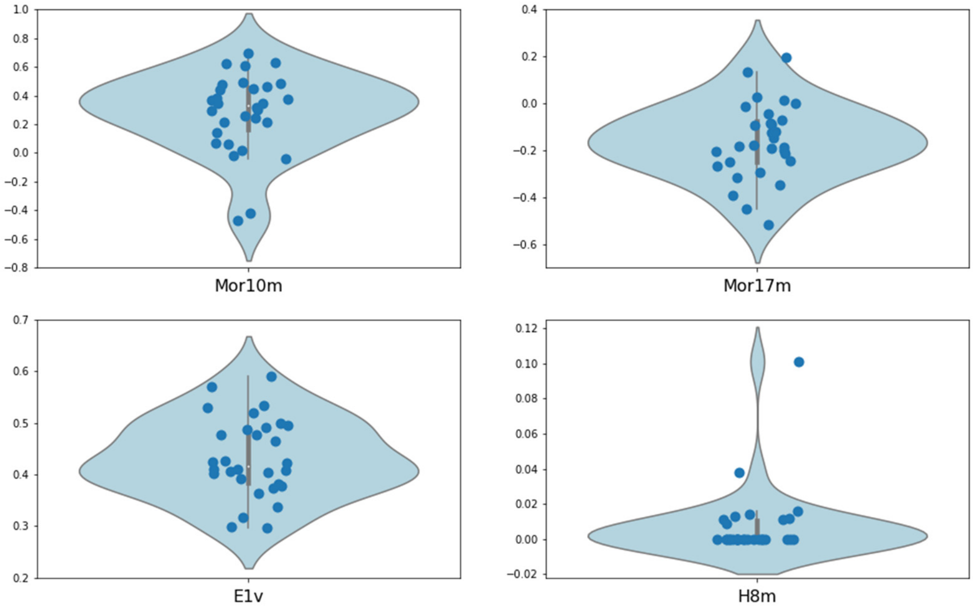

Figure S5. Density distribution of the descriptors values for each variable in a violin plot for the QPON1 model.

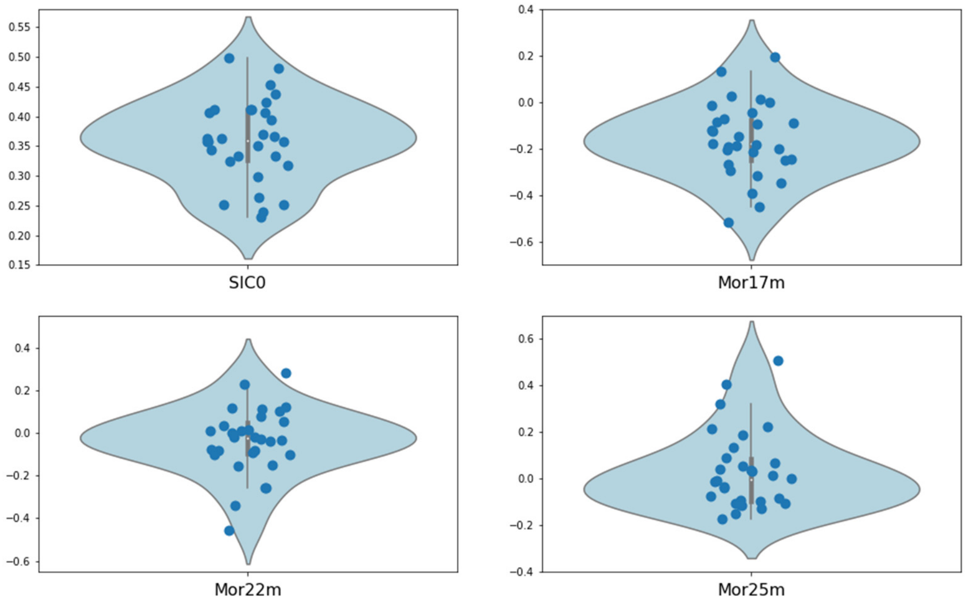

Figure S6. Density distribution of the descriptors values for each variable in the RPON1 model (Plot generated using matplotlib and seaborn library of Python).

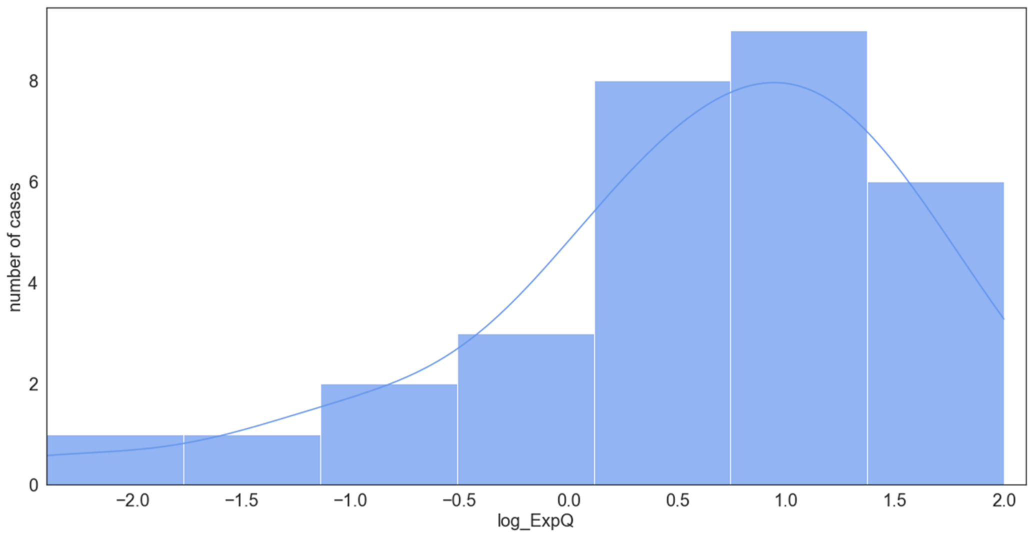

Figure S7. Histogram distribution for QPON1 model based on response variable (rate of reaction).

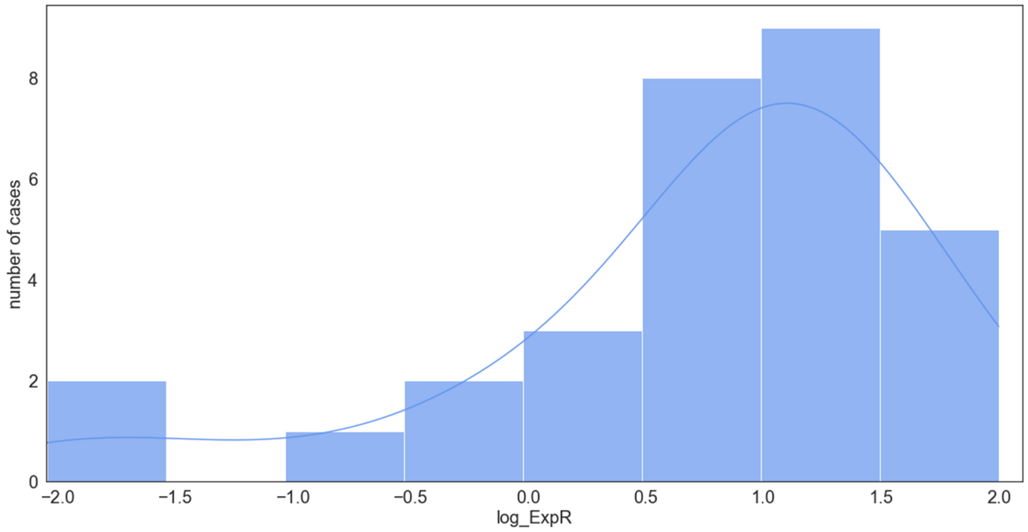

Figure S8. Histogram distribution for RPON1 model based on response variable (rate of reaction).
